# Leukoreductive response to the combination of sorafenib and chemotherapy in hyperleukocytosis of *FLT3*-ITD mutated pediatric AML

**DOI:** 10.3389/fped.2022.1046586

**Published:** 2022-11-09

**Authors:** Franziska Schmidt, Miriam Erlacher, Charlotte Niemeyer, Dirk Reinhardt, Jan-Henning Klusmann

**Affiliations:** ^1^ Pediatric Hematology and Oncology, Hannover Medical School, Hannover, Germany; ^2^ Department of Pediatrics and Adolescent Medicine, Division of Pediatric Hematology and Oncology, University Medical Center Freiburg, Faculty of Medicine, University of Freiburg, Freiburg, Germany; ^3^ Clinic for Pediatrics III, University Hospital Essen, Essen, Germany; ^4^ Department of Pediatrics, Pediatric Hematology and Oncology, Goethe University Frankfurt, Frankfurt, Germany

**Keywords:** hyperleukocytosis, AML, *FLT3*-ITD, sorafenib, leukostasis, oncologic emergencies

## Abstract

Twelve to 22% of pediatric acute myeloid leukemia (AML) patients present with hyperleukocytosis, which is one of the main risk factors of early death due to its clinical complications: leukostasis, causing pulmonary or central nervous system injuries, tumor lysis syndrome, and disseminated intravascular coagulation. Sorafenib is a multi-kinase inhibitor that blocks the Fms-Related Tyrosine Kinase 3 receptor (*FLT3*) in AML patients with a *FLT3*-internal tandem duplication (*FLT3*-ITD), leading to a reduction of proliferation. Here we report four *de novo* diagnosed or relapsed pediatric *FLT3*-ITD–positive AML patients with hyperleukocytosis, which were treated with sorafenib in combination with cytoreductive chemotherapy prior to the start of the induction phase. We observed a fast reduction of white blood cells in peripheral blood and bone marrow. This resulted in a rapid clinical stabilization of the patients. Adverse side effects—such as dermatologic toxicity, elevation of transaminases and hypertension—occurred but were mild and inductive chemotherapy could be started in parallel or subsequently. This implies sorafenib as a safe and effective treatment option in combination with chemotherapy during cytoreductive prephase for children with this life-threatening condition.

## Introduction

Twelve to 22% of pediatric acute myeloid leukemia (AML) patients present with hyperleukocytosis at diagnosis or relapse (1), defined as more than 100 × 10^9^/L white blood cells (WBC). Even with best supportive care, hyperleukocytosis is one of the main risk factors for early death in AML due to leukostasis, causing pulmonary and central nervous system (CNS) injuries, or disseminated intravascular coagulation. Creutzig et al. reported that a WBC count higher than 200 × 10^9^/L increases the risk of death within the first 2 weeks of therapy from 2.4% to 16.9% (2). Furthermore, the risk of nonresponse is higher. To prevent lethal complications, fast and effective leukoreduction is intended. However, tumor lysis syndrome in children (3, 4) as well as its clinical complications (renal dysfunction, cardiac arrhythmia, seizures), induced by too aggressive cytoreduction should be avoided as well. Therefore, intensive supportive care to prevent bleeding, leukostasis and kidney failure is essential. The use of hydroxyurea, low-dose chemotherapy or leukapheresis, could not substantially reduce early mortality in hyperleukocytosis (1), but represent to date the standard approaches. The BFM study group recommends besides intensive, intravenous hydration and hyperurecaemia prophylaxis the treatment with cytarabine (starting with 20–40 mg/m^2^ i.v./s.c. followed by continues infusion with 100 mg/m^2^/24 h). Leukapheresis or exchange transfusion is only recommended for patients with WBC of more than 200 × 10^9^/L or AML FAB M4/M5 (5). Other patients do not seem to benefit from this procedure. Limitations of leukapheresis can often be the requirement of technical expertise and technical problems in small children (6).

One group of AML patients that has an enhanced risk of hyperleukocytosis, are patients with a mutation in the juxtamembrane domain, called internal tandem duplication, of Fms-Related Tyrosine Kinase 3 (*FLT3*-ITD) (7). This mutation causes an independent and constitutive activation of the *FLT3*-receptor due to autophosphorylation, leading to aberrant proliferation and block of apoptosis (8). 12% of pediatric AML patients carry this mutation, whereas the prevalence increases from infants to adolescents (9). In children with AML harboring a mutant *FLT3*-ITD, initial WBC is more often elevated above 50 × 10^9^/L than in wild type (60.1% vs. 34.7%) (10). The rate of *FLT3*-ITD mutations in patients with hyperleukocytosis is high, ranging between 27% and 62% (7, 11, 12).

Sorafenib is a multi-tyrosine kinase inhibitor. One of the kinases being inhibited by sorafenib is *FLT3* (13). In a murine AML cell line, Zhang et al. could show that the effect of sorafenib is 1,000- to 3,000-fold stronger against mutated than wild type *FLT3* (14).

Several studies showed efficacy of sorafenib in the treatment of AML leading to fast remission in combination with chemotherapy as well as monotherapy (15–17). Therefore, sorafenib became part of the AML BFM 2012 trial for children with *FLT3*-ITD/TKD mutation during induction, consolidation and re-intensification (18). A tolerable toxicity profile was reported, consisting of fatigue, hand foot skin reaction, hypertension, elevated transaminases and mild myelosuppression (19). In cases, when sorafenib was used as treatment of relapsed or refractory AML, a fast and efficient leukoreduction has been reported (20–22), suggesting that it might be of clinical benefit in the management of hyperleukocytosis, even in cases where the *FLT3*-ITD status in unknown.

## Patients and methods

### Study design

We conducted a retrospective analysis of four pediatric AML patients presenting with hyperleukocytosis that were reported to the AML-BFM study group and who were treated with sorafenib prior to the start of the induction phase based on the decision of the treating doctor or local hospital. Clinical condition, complete blood cell (CBC) counts, serum electrolytes and coagulation parameters and parameters of tumor lysis syndrome before, during and after treatment with sorafenib were analyzed. Response to treatment was evaluated depending on the reduction of WBC in peripheral blood after 72 h. To identify and analyze mutations in the *FLT3* gene in AML patients, a combined NGS and a fragment assay that detects and quantifies selected mutations of the *FLT3* gene was used.

### Patients

The patients, diagnosed with AML and hyperleukocytosis, were reported to the AML-BFM study group. They were *FLT3*-ITD positive and presented with a hyperleukocytosis and were in a clinically critical state, whereas one of them was *de novo* diagnosed and three of them presented with relapsed or refractory AML. The *FLT3*-ITD status was not known at the start of treatment. In relapsed/refractory AML patients molecular genetics, including the *FLT3*-ITD status, was reassessed.

### Conception of the treatment schedule

All four patients received sorafenib for 3–4 days during the cytoreductive phase. Either sorafenib was given as monotherapy or additionally at the start of chemotherapy at a dose of 2 × 84–2 × 200 mg/m^2^/day. The specific treatment was dependent on the patient's condition or pre-treatments. The cytoreductive treatment was accompanied by supportive care to control metabolic parameters. This included rasburicase or allopurinol, intensive, intravenous hydration, urine alkalization, correction of coagulopathy and treatment of fever and/or infection. Patients were monitored for tumor lysis syndrome using the Cairo-Bishop definition for children. Laboratory tumor lysis syndrome was considered when meeting two or more of the following criteria: hyperuricaemia (uric acid ≥476 µmol/L or 25% increase from baseline), hyperkaliaemia (potassium ≥6.0 mmol/L or 25% increase from baseline), hyperphosphatemia (phosphate ≥2.1 mmol/L or 25% increase from baseline), hypocalciemia (calcium ≤1.75 mmol/L or 25% decrease from baseline) (4). On occurrence of side effects continuation of sorafenib was reevaluated. Treatment or prophylaxis of hand foot skin reaction was at the discretion of the local physician.

## Results

**Figure 1:**
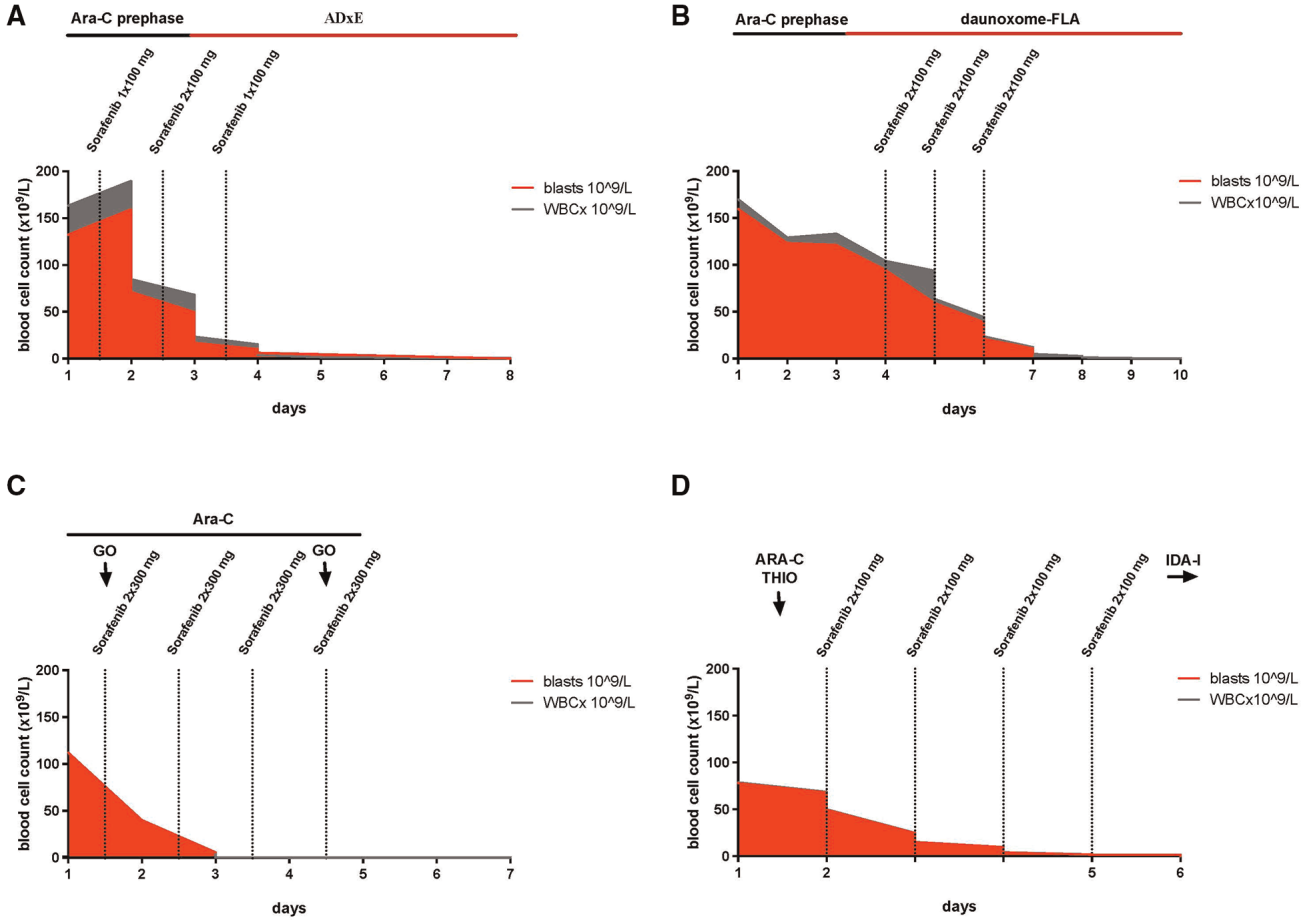
Timelines, illustrating the reduction of white blood cells during treatment with sorafenib and depicting the timing of chemotherapeutic treatment in relation to sorafenib. (**A**) Patient #1: Cytarabine (prephase Ara-C) was started together with sorafenib on day 1 with 1 × 100 mg, increasing the dose to 2 × 100 mg on day 2 and 1 × 200 mg on day 3, on day three induction with intravenous therapy with cytarabine, liposomal daunorubicin and etoposide and intrathecal therapy with cytarabine, methotrexate and prednisolone (ADxE). (**B**) Patient #2: Due to an inadequate response to the cytoreductive prephase with cytarabine (100 mg/m^2^/day continuous infusion) for 3 days (prephase Ara-C), sorafenib (2 × 100 mg/day) was administrated parallel to daunoxome-FLA consisting ofintravenous therapy with liposomal daunoxome, fludarabine, cytarabine and intrathecal therapy with cytarabine, methotrexate and prednisolone for 3 days. (**C**) Patient #3: For 4 days, sorafenib (2 × 300 mg/day) was administrated in combination with cytarabine, 500 mg/m^2^/day, (Ara-C), and gemtuzumab ozogamicin, 2 × 5 mg, (GO). (**D**) Patient #4: On day 2 of treatment with cytarabine, 37.5 mg/m^2^, (ARA-C) and thioguanine, 33 mg/m^2^, (THIO), the treatment was expanded by sorafenib (2 × 100 mg/day) for 4 days, followed by cytarabine without idarubicine (IDA-I).

Between December 2013 and April 2016 four patients with AML and hyperleukocytosis that were treated with sorafenib were reported to the AML-BFM study group. The patients were in a clinically critical state. One of them was *de novo* diagnosed and three of them presented with relapsed or refractory AML. The patients had hyperleukocytosis with an average WBC count of 131 × 10^9^/L (range 80–170 × 10^9^/L) and an average percentage of blasts of 94% (range 81%–100%) before start of therapy. Since the disease status differed between the patients, the course of treatment is presented per patient (Figure 1; Supplementary Table S1).

### Patient #1

The first patient was a 5-year-old boy, diagnosed with *de novo* AML FAB M5, *FLT3*-ITD positive with an *FLT3*-ITD allele ratio of 0.88, trisomy 8 and *MYC* mutation. At the time of diagnosis, he had a WBC count of 163 × 10^9^/L with 81% blasts, isolated petechiae and a disseminated intravascular coagulation (DIC) score of 6 according to the modified criteria of the International Society on Thrombosis and Haemostasis (ISTH) (23, 24). Initially, the patient presented with hepatosplenomegaly, elevated lactate dehydrogenase (LDH) (1,226 UI/L) and aspartate aminotransferase (AST) (113 UI/L). CNS status was negative. Prephase with cytarabine (starting with single doses of 20 and 40 mg/m^2^, then continuous infusion of 100 mg/m^2^/24 h for 48 h) was initiated. One the same day and before receiving the results of molecular diagnostics, sorafenib was administered for 72 h starting at a dose of 100 mg/12 h for 48 h followed by 200 mg/24 h for the following. After 72 h, WBC declined to 3.5 × 10^9^/L. Cytoreductive prephase was followed by the induction protocol *ADxE* according to the recommendations of the AML-BFM 2012 registry (intravenous therapy with cytarabine, liposomal daunorubicin and etoposide and intrathecal therapy with cytarabine, methotrexate and prednisolone). The first dose of liposomal daunorubicin overlapped with the sorafenib treatment. When WBC normalized sorafenib was terminated. On the day of admission, he was diagnosed with influenza B (by polymerase chain reaction). He showed fever but no other clinical signs of infection. On the same day, treatment with oseltamivir was started as well as an intravenous antibiotic treatment with piperacillin, tazobactam and tobramycin. During the cytoreductive prephase, the patient developed a transient hypocalcemia with a minimum serum calcium of 1.74 mmol/L, hyperphosphatemia (2.18 mmol/L), and 25% increase of the potassium level over baseline (max. 5.2 mmol/L), which just barely meets the criteria of a laboratory tumor lysis syndrome in children (4, 25) (Supplementary Table S1). With supportive measures, the laboratory changes improved within 1 day and no clinical symptoms of a tumor lysis syndrome were reported. Renal parameters remained stable, a decline of WBC to less than 0.5 × 10^9^/L was achieved (Figure 1A). The fever persisted spontaneously and LDH and transaminases declined. Subsequent to the treatment with sorafenib, an echocardiography showed no abnormalities in cardiac function.

### Patient #2

The second patient, a 16-years-old male, was diagnosed with AML FAB M1 with Auer rods, mutation in *WT1* and *FLT3*-ITD, trisomy 8 and a deletion in 2p. He had a relapse 1 year after allogeneic hematopoietic stem cell transplantation (HSCT) with 170 × 10^9^/L WBC and 94% blasts in the peripheral blood. He showed laboratory findings of DIC (DIC-score of 6) as well as petechiae at his legs but without complications of bleeding. Due to an inadequate response (WBC 135 × 10^9^/L) to the cytoreductive prephase with cytarabine (100 mg/m^2^/day continuous infusion) for 3 days and coagulopathy (D-dimere >30 mg/L, fibrinogen 2.67 g/L), sorafenib with a dose of 1 × 200 mg/day for 3 days was started together with the reinduction therapy *daunoxome-FLA* of the Relapsed AML 2009 registry (intravenous therapy with liposomal daunoxome, fludarabine, cytarabine and intrathecal therapy with cytarabine, methotrexate and prednisolone). 72 h after the start of sorafenib, WBC decreased to 0.2 × 10^9^/L (Figure 1B). At diagnosis, he presented with fever and a CRP of 69.7 mg/L without a focus of infection. When fever continued, antibiotic and antifungal therapies were escalated and oxygen therapy with up to 4 L/min was required. Later on, respiratory syncytial virus was detected and he was treated with ribavirin. With a history of transposition of the great arteries following arterial switch operation, cardiac function was controlled by echocardiography and showed a sufficient cardiac output. On the second day of treatment with sorafenib, LDH increased to a maximum of 2,997 UI/L, AST was slightly elevated (77 UI/L) and an abdominal ultrasound showed no enlargement of the liver and only marginal increase in spleen size. During administration of sorafenib, the patient barely met the criteria of laboratory tumor lysis syndrome with an increase of potassium as well as urine acid of more than 25% of baseline to a maximum of 5.0 mmol/L and 132 µmol/L, respectively, which is still in the limit of normal (Supplementary Table S1). However, no clinical features of a tumor lysis syndrome were observed. Values decreased during intensified hydration and treatment with rasburicase, later with allopurinol. On the last day of the treatment with sorafenib, the patient developed exanthema on the hands and feet corresponding to hand foot skin reaction. During the following days, the skin reactions improved and only got worse later during the therapy, when sorafenib was restarted between the first and the second course of chemotherapy.

### Patient #3

The third patient was a 17-year-old female, with refractory AML FAB M1/2, *FLT3*-ITD, 8 months after initial diagnosis. She presented with hyperleukocytosis of 112 × 10^9^/L WBC with 100% blasts. In addition to the treatment with cytarabine (500 mg/m^2^/day) for 4 days and gemtuzumab ozogamicin (5 mg on day 1 and 4) she received sorafenib 2 × 300 mg/day. WBC decreased to 0.3 × 10^9^/L within 72 h (Figure 1C). LDH rose to a maximum of 2,060 UI/L on the first day of administration with sorafenib. The criteria for laboratory tumor lysis syndrome were not fulfilled (Supplementary Table S1). Under antiemetic therapy with ondansetron and dimenhydrinate, chemotherapy was tolerated well. Having fever already before admission, she developed pneumonia, which required anti-infectious treatment and oxygen therapy with 5 L/min. During the cytoreductive phase, hypertension was treated with amlodipine and hydrochlorothiazide as well nifedipine and dihydralazine as needed. Cardiac function after termination of cytoreductive therapy was normal. A hematoma, resulting from complications during the implantation of a central venous catheter, aggravated the decrease of hemoglobin (hemoglobin 6.2 mg/dl) and required several transfusions of red blood cells, thrombocytes and fresh frozen plasma. Two weeks after cytoreductive therapy had been started, bone marrow aspiration showed a reduction of blasts from 94% to 15%. A third dose of gemtuzumab ozogamicin was administered. Sorafenib orally 2 × 300 mg was given until conditioning regimen for stem cell transplantation was started.

### Patient #4

The fourth patient was an 11-year-old boy with AML FAB M4, mutations in *WT1* and *FLT3*-ITD (allele ratio of 0.54), who relapsed during first-line therapy with 50% blasts in peripheral blood before the last of five courses (AML-BFM 98 trial). The therapy of the relapse was induced with a course of idarubicin, fludarabine, high dosage cytarabine and G-CSF (IDA-FLAG), to which he did not respond, presenting a WBC count of 80 × 10^9^/L and 99% blasts in peripheral blood. An off study treatment to achieve cytoreduction was started. On the first day, he received cytarabine (37.5 mg/m^2^) and thioguanine (33 mg/m^2^). On the second day, sorafenib with a dose of 2 × 100 mg/day was added for 4 days due to inadequate response. The WBC decreased to 1.9 × 10^9^/L, 72 h later (Figure 1D). On the third day LDH increased to 2,243 IU/L and alanine transaminase (ALT) to 416 IU/L but declined the next day, when WBCs fell below the level of normal. The level of potassium and phosphate where within the norm (Supplementary Table S1). Under treatment with rasburicase urin acid showed an increase over 25% but maximum level of 89 µmol/L. Parallel to cytoreduction, intrathecal therapy with cytarabine, prednisolone and methotrexate was initiated. Even though cytoreductive treatment of hyperleukocytosis was effective, the subsequent antileukemic treatment could not achieve a remission and the patient succumbed 3 months later.

## Discussion

We present four different pediatric cases of *FLT3*-ITD-positive AML and hyperleukocytosis that were treated with sorafenib during their cytoreductive prephase or—in one case—after inadequate response to the cytoreductive prephase in parallel to induction. In all four patients, WBC fell within 72 h after start of sorafenib administration to or below the level of normal (Figure 1). The patients had no signs of neurologic or renal toxicity or liver failure. But all patients experienced anemia and thrombocytopenia, without developing massive bleeding but making transfusion of blood components inevitable. The changes in electrolytes—despite massive cell reduction—were mild and only two patients just about met the criteria of a laboratory tumor lysis syndrome, which normalized under intensified supportive care. One patient developed hand foot skin reaction, which is very common during sorafenib treatment. This suggests that sorafenib administration for several days as cytoreductive prephase in children with AML and hyperleukocytosis may be effective and well-tolerated, even if administered in combination with cytarabine, liposomal daunorubucin or gemtuzumab ozogamicin. However, prospective clinical trials are required to determine the role of sorafenib in the treatment of hyperleukocytosis in AML.

A relevant factor in considering a treatment with sorafenib is the high incidence of adverse events that occur during hyperleukocytosis. On the other hand, pediatric patients suffer more often from toxicity due to sorafenib, probably due to the higher conversion to active n-oxide metabolites (16). Still the short treatment interval and the life-threating condition counterbalance this risk and seem to rectify the inclusion of sorafenib to the cytoreductive if *FLT3*-ITD-positive AML with hyperleukocytosis. One of the most common dose-dependent adverse effects of sorafenib is dermatologic toxicity, especially when used in combination with chemotherapy (15, 16, 19). In fact, hand foot skin reaction was observed in one of four patients. However, as the patient simultaneously received high dose cytarabine, it is unknown which of the two drugs caused this reaction. Still, the symptoms aggravated again, when sorafenib was restarted, implying that sorafenib at least influenced the erythema. Elevation of transaminases is another common adverse event (16, 19), which occurred in two of our patients. Only in one patient, transaminase elevation was classified as grade 3 (NCI CTCAEv3.0) (26). As ALT decreased the following day, no discontinuation of drug administration became necessary (19). The first patient showed a 2-fold elevated AST already at diagnosis, suggesting that this was related to the leukemia itself. The third patient showed manageable hypertension under administration of sorafenib, so that discontinuation of sorafenib was not necessary. Hypertension is an infrequently reported side effect of sorafenib in children (16, 19) even though it is common in adults (27) and mostly manageable with the use of angiotensin-converting enzyme inhibitors and beta-blockers. Tarlock et al. reported two patients, who experienced adverse cardiac events during treatment with sorafenib, whereas one of them received chemotherapeutics causing cardiac side effects in parallel to administration of sorafenib (15). Showing an elevated incidence for cardiac ischemia in adults as well (27), cardiac function of patients, presenting with a history of cardiac diseases or receiving drugs that can cause cardiac dysfunction, should be controlled regularly. Our patients showed no abnormalities in cardiac function. Targeted therapies—including sorafenib—in the initial phase of treatment have the potential to increase the incidence of a tumor lysis syndrome (28–31). In our cohort, the patients received preventive, supportive care, consisting of intensified intravenous hydration, hypouricemic agents and urinary alkalization, which precented massive or clinically relevant tumor lysis syndrome. We anticipate that this can be attributed to a stronger effect of sorafenib on cell proliferation than on apoptosis in AML with hyperleukocytosis (14, 32).

Our patients were treated with a dose of 2 × 84–2 × 200 mg/m^2^/day. Based on a phase I study of the Children's Oncology Group, the recommended dose of sorafenib for children with leukemia is 150 mg/m^2^ every 12 h (19). Inaba et al. recommended the identical dose for the use in combination with cytarabine or clofarabine (16). If sorafenib is considered as a treatment option in future cases of hyperleukocytosis this could serve as a reference dosage. The reported patients presented with different disease status (initial diagnosis or relapse), risk profile or pre-treatment. Therefore, combinations of sorafenib with the different chemotherapeutic agents were required. All combinations showed an effective leukoreduction. Since several studies show a limited single-agent activity (33–35) our study does recommend sorafenib in combination with chemotherapy for effective and safe cytoreduction in case of hyperleukocytosis. Following that, an intensive multiagent induction chemotherapy is indispensable to achieve remission.

Sorafenib is less effective in *FLT3* wild type (WT) compared to *FTL3*-ITD (14, 17). In contrast to *FLT3*-WT, *FLT3*-ITD enhances the interactions of sorafenib at the ligand binding site (14). Furthermore, AML cell lines with wild type *FLT3* show a paradox phosphorylation and thereby activation of the *ERK2 (MAPK1)* pathway, possibly explaining the resistance of sorafenib in *FLT3*-WT AML cell lines (36). These findings make effective treatment of hyperleukocytosis with sorafenib more likely in *FLT3*-ITD than in *FLT3*-WT. However, results of mutation analysis are not always available in the first hours after diagnosis. Largeaud et al. recently analyzed the genomic landscape of adult AML patients with hyperleukocytosis. The study revealed an average of four mutations per patient, whereby “drug-actionable” mutations were detected in 113 of 154 patients; 96 of them carried mutations in *FLT3* (62.3%) (12). Thus, seeing hyperleukocytosis as a severe life-threatening condition in the progression of AML with a high frequency of activating mutations in signaling cascades, particularly in *FTL3* (12), and showing a favorable toxicity profile, addition of sorafenib should be considered early during hyperleukocytosis and—if necessary—already before receiving the mutational analysis. Our patients were treated with sorafenib for cytoreduction as it was already part of the AML BFM 2012 trial for children with *FLT3*-ITD/TKD mutation during induction, consolidation and re-intensification (18). However, with gilterinib and midostaurin two drugs were recently approved for the treatment of *FLT3*-mutated AML (37, 38). Both are under investigation evaluation in children as a single agent or in combination with chemotherapy in newly diagnosed or relapsed/refractory AML (NCT04240002, NCT03591510 and NCT00866281). In a prospective trial of Stone et al., midostaurin showed a blood blast reduction in 70%, and a bone marrow blast reduction of over 50% in 6 of 20 patients. Nevertheless no complete remission was achieved by monotherapy with midostaurin (39). Still, both substances could potentially be used as an alternative to sorafenib in children with AML and hyperleukocytosis.

In summary, on the basis of our here presented experience, we think that sorafenib—or other *FLT3* inhibitors—in combination with chemotherapy is an adequate treatment option for cytoreduction in AML *FLT3*-ITD hyperleukocytosis.

## Data Availability

The original contributions presented in the study are included in the article/**Supplementary Material**, further inquiries can be directed to the corresponding author/s.
