# Supplementary material for: Leukoreductive response to the combination of sorafenib and chemotherapy in hyperleukocytosis of FLT3-ITD mutated pediatric AML
Source: Front Pediatr. 2022 Nov 9;10:1046586. doi: 10.3389/fped.2022.1046586 (PMC9681922; doi:10.3389/fped.2022.1046586)
Supplement: Supplementary file 1 [file Datasheet1.pdf]

Supplementary Material

Table S1: Tumor lysis parameters

Baseline value, defined as value before or at the start of the therapy with sorafenib (BL) and maximum level (MAX) or minimum level (MIN) of specified electrolytes (potassium, phosphorus, calcium) and uric acid within the time of cytoreduction under sorafenib administration. Values are taken from serum blood samples, except potassium levels, which are measures in serum (s-) and blood gas (BG), For patient #3, base lines of potassium and phosphorus given only qualitatively, normal range (NR), calcium was not defined changes within the calcium level were not reported (ND) in patient 3. Uric acid was not defined (ND) for patient 4.

	s- or BG-potassium [mmol/l]		s-phosphate [mmol/l]		s-calcium [mmol/l]		s-uric acid [µmol/l]	
**Patient**	**BL**	**MAX**	**BL**	**MAX**	**BL**	**MAX**	**BL**	**MAX**
**#1**	3.50	5.20	1.60	2.18	2.05	1.74	ND	ND
**#2**	3.70	5.00	1.28	2.09	2.25	1.86	< 14	132.00
**#3**	2.40	4.50	<0.1	1.09	2.30	1.74		< 14
**#4**	NR	5.00	NR	1.50	-	-	< 5.9	89.00
